# On the artefactual parasitic eubacteria clan in conditioned logdet phylogenies: heterotachy and ortholog identification artefacts as explanations

**DOI:** 10.1186/1471-2148-10-343

**Published:** 2010-11-09

**Authors:** Ajanthah Sangaralingam, Edward Susko, David Bryant, Matthew Spencer

**Affiliations:** 1 Centre of Haemato-Oncology, Institute of Cancer, Bart's and the London School of Medicine (QMUL), Charterhouse Square, London EC1M 6BQ, UK; 2 Department of Mathematics and Statistics, Dalhousie University, Halifax, Nova Scotia, B3H 3J5, Canada; 3 Department of Mathematics and Statistics, University of Otago, P.O. Box 56, Dunedin, New Zealand; 4 School of Environmental Sciences, University of Liverpool, Liverpool L69 3GP, UK

## Abstract

**Background:**

Phylogenetic reconstruction methods based on gene content often place all the parasitic and endosymbiotic eubacteria (parasites for short) together in a clan. Many other lines of evidence point to this parasites clan being an artefact. This artefact could be a consequence of the methods used to construct ortholog databases (due to some unknown bias), the methods used to estimate the phylogeny, or both.

We test the idea that the parasites clan is an ortholog identification artefact by analyzing three different ortholog databases (COG, TRIBES, and OFAM), which were constructed using different methods, and are thus unlikely to share the same biases. In each case, we estimate a phylogeny using an improved version of the conditioned logdet distance method. If the parasites clan appears in trees from all three databases, it is unlikely to be an ortholog identification artefact.

Accelerated loss of a subset of gene families in parasites (a form of heterotachy) may contribute to the difficulty of estimating a phylogeny from gene content data. We test the idea that heterotachy is the underlying reason for the estimation of an artefactual parasites clan by applying two different mixture models (phylogenetic and non-phylogenetic), in combination with conditioned logdet. In these models, there are two categories of gene families, one of which has accelerated loss in parasites. Distances are estimated separately from each category by conditioned logdet. This should reduce the tendency for tree estimation methods to group the parasites together, if heterotachy is the underlying reason for estimation of the parasites clan.

**Results:**

The parasites clan appears in conditioned logdet trees estimated from all three databases. This makes it less likely to be an artefact of database construction. The non-phylogenetic mixture model gives trees without a parasites clan. However, the phylogenetic mixture model still results in a tree with a parasites clan. Thus, it is not entirely clear whether heterotachy is the underlying reason for the estimation of a parasites clan. Simulation studies suggest that the phylogenetic mixture model approach may be unsuccessful because the model of gene family gain and loss it uses does not adequately describe the real data.

**Conclusions:**

The most successful methods for estimating a reliable phylogenetic tree for parasitic and endosymbiotic eubacteria from gene content data are still ad-hoc approaches such as the SHOT distance method. however, the improved conditioned logdet method we developed here may be useful for non-parasites and can be accessed at http://www.liv.ac.uk/~cgrbios/cond_logdet.html

## Background

Phylogenetic reconstruction methods based on sequence data have difficulty in accounting for events such as genome fusion and horizontal gene transfer that occur during evolution [1]. As a result, phylogenies based on such data may not represent organismal lineages. One possible solution is to estimate phylogenies from the presence/absence of gene families in completely sequenced genomes [2]. However, differences in genome size may bias the results of gene content methods. These differences result from variation in gene family gain and loss rates between lineages. A recent model which estimates the evolutionary history of gene content shows that such heterogeneity in gain and loss rates is common throughout the tree of life [3].

SHOT (SHared Ortholog and gene order Tree reconstruction tool, [4]) is a method for estimating gene content phylogeny which avoids some of the problems with genome size variation by ignoring shared absences. However, it is not based on any specific model of evolution. Thus, although sensible phylogenies are often estimated [4], distances derived from SHOT are not tree-additive. The method is therefore inconsistent in the statistical sense. For many parameter settings of standard evolutionary models, SHOT is not guaranteed to estimate the phylogeny correctly even with infinite data [5]. It is therefore difficult to trust any results from SHOT that are not supported by methods with better statistical properties, and a consistent statistical method for estimating phylogenies from gene content data remains desirable.

Logdet distances were developed to deal with biases in tree estimation caused by variation in nucleotide composition among sequences [6]. Logdet distances from gene content data might be a good way to deal with varying genome size [7]. In a highly-cited and controversial [8-12] paper, Rivera & Lake [13] used trees based on gene content logdet distance to support a new theory about the origins of eukaryotes. They dealt with the problem of unobservable gene families (such as those absent everywhere) by analyzing only the gene families found in an arbitrary 'conditioning genome'. Their approach is known as conditioned genome reconstruction. A refinement of their method which avoids the arbitrary choice of one conditioning genome (which can systematically bias the results: [14,15]) is to estimate a distance matrix using each possible conditioning genome in turn, and combine the results using a supertree method [5]. This refined approach is consistent and outperforms the original method.

Conditioned logdet distances have been applied to bacterial species from the COG database [5] and give a mostly-plausible phylogeny. However, the intracellular parasitic and endosymbiotic eubacteria (parasites for short) form a clan in this tree. The same is true of other methods for estimating phylogenetic trees from gene content data [16], with the exception of SHOT [4]. Other lines of evidence suggest that the parasitic lifestyle has arisen independently many times [17]. A sequence-based tree estimated from a large number of orthologs which are unlikely to have been laterally transferred does not contain a parasites clan [18]. It is therefore likely that the parasites clan is an artefact.

There are at least two possible underlying causes for the artefactual parasites clan. First, it could be an ortholog identification artefact. For example, differences in definitions of orthologs have been shown to affect phylogenetic reconstruction methods based on gene content. Gene families assembled with a stricter homology criterion (giving smaller gene families) resulted in better estimates of prokaryotic phylogeny than those assembled with less strict criteria [19]. We will test the idea that the parasites clan is an ortholog identification artefact (of some unknown kind) by applying methods based on conditioned logdet distance to three different gene content databases, constructed using different methods and therefore likely to be subject to different biases. If the parasites clan appears in trees from all three databases, it is unlikely to be an ortholog identification artefact.

Second, the parasites clan could be a result of differences in the rates of gene gain and loss amongst parasites and non-parasites in a subset of gene families. Some parasites rely on their hosts to perform certain functions [20]. Corresponding genes in the parasite are then less important, and may experience accelerated loss rates. In contrast, those gene families whose functions cannot be performed by the host are unlikely to have an accelerated rate of loss in parasites. The consequence is that there may be a subset of gene families with much higher probabilities of absence in parasites than other genomes. This form of heterotachy is known to cause problems in phylogenetic reconstruction [21], and cannot be dealt with using logdet distances [6].

Mixture models have been proposed as a way of dealing with heterotachy [22]. In mixture models for gene family gain and loss, gene families are divided automatically into two classes. Essential genes have the same rates of loss in parasites and non-parasites. Non-essential genes have an accelerated rate of gene loss in parasites. However, existing mixture models are too simple to deal with other causes of variation in genome size, for which logdet distances may be suitable. We therefore consider a hybrid method, in which we use a mixture model to calculate conditioned logdet distances separately for each class. If this method results in correct placement of the parasites, it would support the idea that the parasites clan is caused by heterotachy. We consider two different mixture models. The first is a simple non-phylogenetic model, which does not require knowledge of the tree topology, but incorrectly assumes that genomes are independent of each other. The second is a phylogenetic mixture model [23], in which we do not assume genomes are independent, but do need to specify a tree topology. Comparing the performance of these methods will establish whether sophisticated phylogenetic mixture models are necessary in order to assign gene families to the essential and non-essential categories. We compare the gene family assignments from the two mixture models, and examine the relationship between COG functional categories and mixture model categories. We also use simulations to evaluate the mixture models in cases where the true tree is known.

In addition to testing the above hypotheses we have addressed some problems that previously limited the usefulness of conditioned logdet distances. In our original implementation of conditioned genome reconstruction [5], non-existent logdet distances sometimes occur, and the taxa involved must be excluded from the tree. We have developed new software that addresses this problem.

We show that the parasites clan is unlikely to be an ortholog identification artefact. Partitioning gene families using the non-phylogenetic mixture model mostly broke up the parasites clan, although overall the resulting trees were not more similar to a reference tree based on 16S rRNA sequences. Thus, although the heterotachy idea remains plausible, we still do not have reliable phylogeny estimation methods based on conditioned logdet distances.

## Methods

Presence/absence data from orthologous gene families for fifty bacterial species were extracted from the COG database [24], which contains information on the distribution of 4873 gene families. We then extracted corresponding presence/absence data for the same species from the TRIBES [25] and OFAM databases [26], which use different methods to identify orthologous genes. In cases where more than one strain from TRIBES and OFAM was a potential match to the strain used in COG, we included all strains. Thus the TRIBES data analyzed contained 67 taxa and 16122 gene families, while the OFAM data contained 67 taxa and 308593 gene families. In COG, orthologs are identified by pairwise comparison using the BLASTPGP program between all protein sequences encoded in all sequenced genomes, and clustered based on triangular patterns of reciprocal best hits. In contrast, TRIBES and OFAM use the Markov Cluster (MCL) algorithm for protein family assignment based on pairwise similarities [27]. Thus, biases introduced by the methods of ortholog identification are likely to be different between COG and the other databases.

The data we used are available to download from http://www.liv.ac.uk/~cgrbios/genome_data.zip

The following twelve taxa were identified as intracellular parasites/endosymbionts, using information from Bergey's Manual of Systematic Bacteriology [28]: *Mycobacterium leprae*; *Buchnera *sp. APS; *Rickettsia prowazekii*; *Rickettsia conorii*; *Chlamydia trachomatis*; *Chlamydia pneumoniae *CWL029; *Treponema pallidum*; *Borrelia burgdorferi*; *Ureaplasma urealyticum*; *Mycoplasma pulmonis*; *Mycoplasma pneumoniae *and *Mycoplasma genitalium*. These assignments were based on an explicit reference in Bergey's manual to an obligate intracellular lifestyle, failure to cultivate in a tissue culture or artificial medium or other evidence of reduced metabolic activities.

### Phylogeny Estimation Using Conditioned Logdet

Presence/absence data on orthologous gene families were extracted into files in PHYLIP format. Conditioned logdet distance matrices and conditioning genome sizes were calculated for each choice of conditioning genome. We used constrained maximum likelihood and pseudocount methods to deal with non-existent distances (Additional file 1, section 1.7). A modified version of BIONJ [29] was used to combine information from all distance matrices into a single tree [5]. Distance matrices were weighted by the inverse of their variances to account for differences in reliability. The source code for calculating conditioned logdet distances and the modified version of BIONJ was written in C. A web server incorporating these methods is available at http://www.liv.ac.uk/~cgrbios/cond_logdet.html

Two hundred bootstrap replicates were run and PHYLIP CONSENSE [30] was used to calculate a majority rule consensus tree.

### Application of non-phylogenetic mixture model

We used a non-phylogenetic mixture model written in R [31] (Additional file 1, section 2) to assign gene families to essential and non-essential categories. We treated genomes as independent, so that gene family presence/absence can be described by a binomial mixture model. We assigned gene families to categories using empirical Bayes.

Distance matrices were calculated separately for each category of gene families as described, and then combined using a weighted sum (Additional file 1, section 3). Trees were estimated as described above. When bootstrapping, we treated the assignments to categories as fixed. PHYLIP CONSENSE was used to calculate a majority rule consensus tree.

### Application of phylogenetic mixture model to COG dataset

The phylogenetic mixture model is a heterogeneous model with two major categories of gene families: essential and non-essential. We model gain and loss of gene families in each of these categories on a rooted tree with a known topology. The gain and loss rates in the non-essential category change at points on the tree where lineages become parasitic or endosymbiotic. In the model, gain and loss rates change at the basal end of the most basal edge having only parasites as descendant leaves. We use maximum likelihood to estimate the gain and loss rates and the proportion of gene families in each category. We then use empirical Bayes methods to assign gene families to categories [23].

We fitted phylogenetic mixture models to the COG data on a 16S rRNA tree as described in [23]. This 16S rRNA tree was treated as the estimate of the true tree (Figure S1, Additional File 2). Estimated trees are compared to this throughout. The best-fitting model (model F in [23]) had two categories of genes (essential and non-essential), each with four gamma rate classes (from slow to fast gain and loss, within the essential and non-essential categories). After using empirical Bayes to assign gene families to combinations of categories and rate classes, we discarded combinations that contained very few gene families, as distance estimates from these combinations are likely to have a very high variance. We calculated conditioned logdet distances for the remaining combinations, and combined them using a weighted sum, as described above. As before, we treated category assignments as fixed when bootstrapping.

### Comparison of COG gene family assignments from the two mixture models

To test whether there was any agreement between the assignments of gene families to the essential and non-essential categories between mixture models, we used a chi-squared test of the null hypothesis of no association between assignments. We also examine the COG functional categories associated with the essential and non-essential categories. If the mixture models are correctly identifying essential gene families, this category should consist of genes whose functions are unlikely to be supplied by the host,

### Application of SHOT algorithm to datasets

PHYLIP SEQBOOT [30] was used to generate two hundred bootstrap resample files for each dataset. For each bootstrap replicate, SHOT distances [4] were calculated using a Perl script. Trees were estimated using BIONJ and a majority-rule consensus obtained using PHYLIP CONSENSE.

### Calculation of 16S rRNA trees for the datasets

16S rRNA sequences for each of the organisms present in each of the three datasets were downloaded from the Ribosomal Database Project release 9.57 [32]. PHYML version 2.4.4 (GTR model with four substitution rate categories and 1000 bootstrap replicates) was used to estimate a maximum likelihood tree for each dataset [33].

### Calculation of Robinson-Foulds (RF) distance

For a pair of trees the RF distance [34] between them is the number of edges present in either one of the trees but not both. For each dataset, we calculated RF distances between the following pairs of trees: conditioned logdet (with and without mixture models) and rRNA: conditioned logdet (with and without mixture models) and SHOT, rRNA and SHOT. PHYLIP was used to calculate distances.

### Simulation studies

100 datasets of 50 genomes and 4873 gene families were simulated as described in [23] using parameter estimates from the best fitting model F. Gene families were classified as above, except that parameters were fixed at their true values, because estimation is very computationally intensive. The phylogeny was estimated for each of the simulated datasets using each of the three methods: conditioned logdet, non-phylogenetic mixture model and the phylogenetic mixture model, as described above.

## Results & Discussion

**Figure 1:**
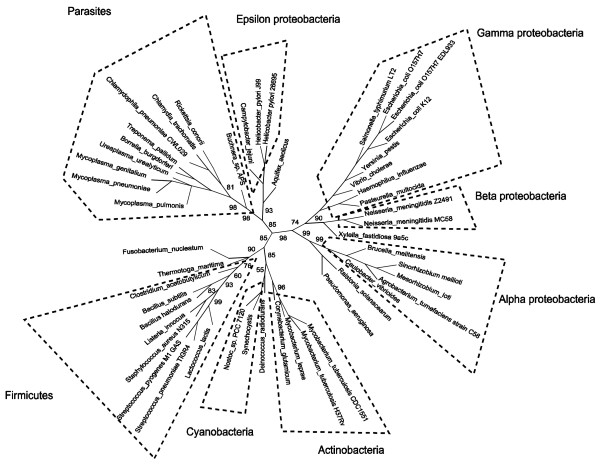
**Unrooted radial cladogram from COG using conditioned logdet distances and modified BIONJ**. Majority rule consensus, 200 bootstrap replicates from PHYLIP CONSENSE. This tree was drawn using Dendroscope [42]. Edge lengths not to scale.

Parasites form a clan with high bootstrap support in conditioned logdet trees from all three databases (COG: Figure 1, 98% bootstrap support. TRIBES: (Additional file 2, Figure S3) 81% bootstrap support. OFAM: (Additional file 2, Figure S7) 89% bootstrap support). Thus, the parasites clan is unlikely to be an ortholog identification artefact.

In other respects, the conditioned logdet phylogeny from the COG database is mostly plausible. The gamma proteobacteria are found together in a clan with 90% bootstrap support, apart from *Xylella fastidiosa *and *Pseudomonas aeruginosa*. Alpha proteobacteria are found together in a clan with 99% bootstrap support. Epsilon proteobacteria are found together in a clan with 93% bootstrap support. However, some taxa are misplaced. *Ralstonia solanacearum *is not found together with the other beta proteobacteria. *Aquifex aeolicus *is placed with the epsilon proteobacteria and *Thermotoga maritima *is placed with the firmicutes. These thermophiles usually occupy a basal position within a bacterial tree.

The conditioned logdet COG tree differs more from the 16S rRNA tree (Additional file 2, Figure S1) than does the SHOT tree estimated from COG data (Additional file 2, Figure S2). In the SHOT tree, all taxa agree well with their 16S placements and have high bootstrap support. In the rest of the results section, we will concentrate on the COG trees, which were generally closer than those from other databases to the 16S trees. Full results from the other datasets are in Additional file 2.

### Effect of applying the non-phylogenetic mixture model to the COG database

**Figure 2:**
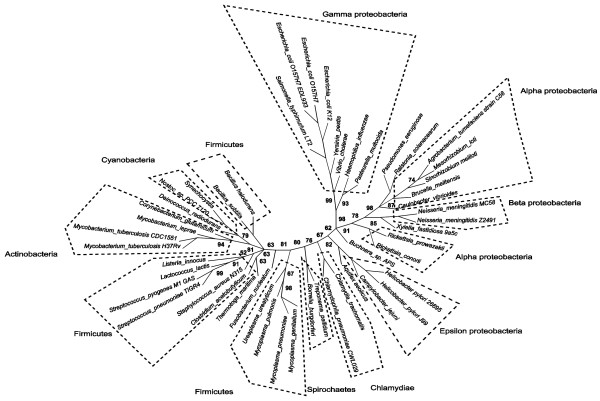
**Unrooted radial cladogram from COG using conditioned logdet distances and non-phylogenetic model**. Majority rule consensus from PHYLIP CONSENSE, 200 bootstrap replicates. This tree was drawn using Dendroscope [42]. Edge lengths not to scale.

Figure 2 shows the results of applying the non-phylogenetic mixture model and conditioned logdet distances to the COG dataset. The parasites are now separated into four different clans. *Rickettsia *is moved towards its correct placement with high bootstrap support, in contrast to the phylogenetic model and conditioned logdet (see below). The *Chlamydiae *are found near the spirochetes *Borrelia burgdorferi *and *Treponema pallidum*. The four parasites that belong to the firmicutes group are: *Mycoplasma genitalium*, *Mycoplasma pulmonis*, *Mycoplasma pneumonia *and *Ureaplasma urealyticum*. These are found close to the other firmicutes, but in a clan which also contains cyanobacteria, actinobacteria, *Thermotoga maritima *and *Fusobacterium nucleatum*. The *Rickettsias *belong to the alpha proteobacteria subgroup. In this tree, they are placed in a clan which includes beta proteobacteria as well as non-parasitic alpha proteobacteria. Thus, overall, the parasites no longer form a clan, but are not in general placed in the groups to which they are usually thought to belong.

### Application of phylogenetic mixture model to the COG data

**Figure 3:**
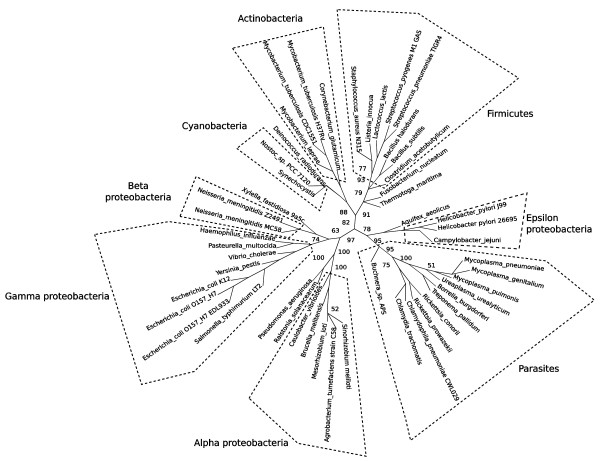
**Unrooted radial cladogram from COG using conditioned logdet distances and phylogenetic model**. Majority rule consensus, 200 bootstrap replicates, from PHYLIP CONSENSE. This tree was drawn using Dendroscope [42]. Edge lengths not to scale.

The tree from the phylogenetic mixture model (Figure 3) contains a parasites clan with 95% bootstrap support. Thus, although the phylogenetic mixture model is based on a more sophisticated underlying model that should capture phylogenetic dependence, it does not perform better than the non-phylogenetic model on these data.

### Analysis of COG functional categories

#### Non-phylogenetic and phylogenetic mixture models

**Figure 4:**
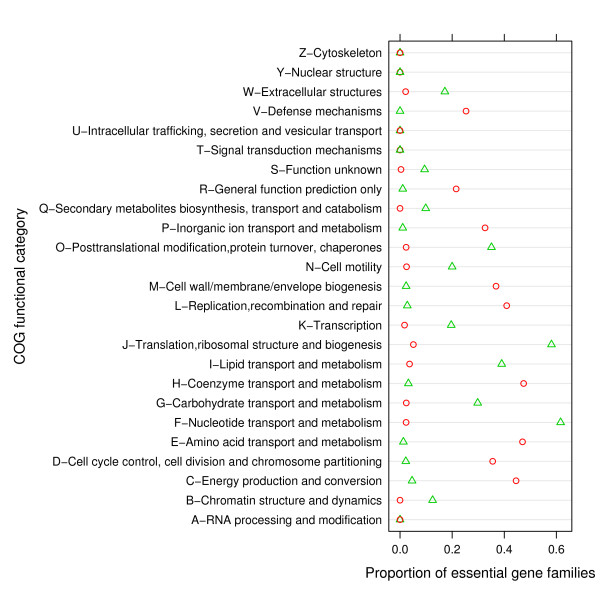
**Proportion of essential gene families among each COG functional category**. Red circles: proportion of essential genes from phylogenetic model, green triangles proportions of essential genes from non-phylogenetic model.

The COG database contains 4873 gene families. In our data, the average number of gene families present is 568 in parasitic genomes and 1568 in non-parasitic genomes. These gene families are distributed among 26 different functional categories. Categories J, A, K, L and B represent functions related to information storage and processing. D, Y, V, T, M, N, Z, W, U and O are cellular processes and signaling categories. C, G, E, F, H, I, P and Q are metabolic processes categories. Categories R and S form the general function prediction only and unknown function groups. Two categories (U: intracellular trafficking, secretion and vesicular transport, and T: signal transduction mechanisms) did not occur in the subset of the COG database we examined). The proportions of essential and non-essential gene families in each COG functional category were found for both the phylogenetic and non-phylogenetic mixture models (Figure 4). Using the non-phylogenetic mixture model, approximately 75% of the gene families were assigned to the non-essential category. Using the phylogenetic model, 95% of the gene families were assigned to the non-essential category. A large proportion of gene families are in the general function prediction only and unknown groups (Figure 4).

The majority of COG categories, including metabolic pathways and information storage and processing, consist mainly of non-essential genes. There are two functional categories for which more genes are assigned to the essential subset by the non-phylogenetic mixture model (Figure 4). These are categories J (translational, ribosomal structure and biogenesis) and F (Nucleotide transport and metabolism). It has been observed that genes from category J have an increased presence in parasitic genomes; therefore they are not easily lost [35]. Our results agree with this observation. It has also been shown that some parasites have a large number of genes encoding proteins involved in the transport and metabolism of nucleotides, again suggesting that genes from this category are not easily lost from parasitic genomes [36].

For both the phylogenetic model and the non-phylogenetic model (Figure 4) categories A (RNA processing and modification), Y (Nuclear structures) and Z (cytoskeleton) are made up entirely of non-essential genes. This suggests that these functions can usually be supplied by the host of a parasite. Genomes of intracellular parasites have lost genes that encode proteins which enhance the efficiency of universal cellular processes such as translation and transcription [17]. Biologically, it makes sense that the functional category for RNA processing and modification is made up of genes that have an increased rate of loss in parasites. Thus, even though neither mixture model greatly improves the placement of parasites in gene content phylogenies, the classification of essential and non-essential gene families from these models seems plausible. This is more or less independent of the phylogenetic performance of the mixture models, because the non-phylogenetic model does not use a phylogeny to classify gene families, while the phylogenetic mixture model uses an input phylogeny (the 16S tree, in this case).

#### Comparison of gene family assignments from the two mixture models

Table 1 shows the number of gene families that were assigned to the same category using each of the two models. There was a significant association between assignments by the two models (χ^2 ^= 26, 1.d.f., *p *= 3.2e-07).

**Table 1:** Number of essential and non-essential gene families found in the same category.

Number of gene families	Non-essential (phylogenetic)	Essential (phylogenetic)
Non-essential (non-phylogenetic)	3356	76

Essential (non-phylogenetic)	1177	64

Agreement table showing the number of essential and non-essential genes found in the same category using both phylogenetic and non-phylogenetic mixture models.

There have been other attempts to assign gene families to essential and non-essential categories. For example, [37] assigned *E. coli *genes to the non-essential and essential categories using experimental evidence and known function. They found a much higher number of non-essential genes compared to essential genes. Their assignments are similar to those from the non-phylogenetic and phylogenetic mixture models, in that we found a higher number of non-essential genes compared to essential genes in each of the four main COG functional categories. The main difference between our work and theirs is that we are using indirect evidence of gain and loss patterns, but are looking at parasites. They are using direct experimental evidence, to look at gene families that are essential to a non-parasite.

Table 2 shows the equilibrium probability of gene family presence for each category of genes and both mixture models. This gives an approximate rather than an exact comparison between models for two reasons. First, for the phylogenetic model, these equilibrium probabilities are only an approximate measure of the expected probabilities, because the model is non-stationary. Second, the phylogenetic model has the same equilibrium probabilities for presence of non-essential gene families in parasites and presence of essential genes in all genomes, while the non-phylogenetic model distinguishes these two probabilities. Nevertheless, there is a clear overall pattern for all data sets and both mixture models, that the probability of non-essential gene family presence is lower in parasitic than non-parasitic gene families.

**Table 2:** Equilibrium probabilities of gene family presence from phylogenetic and non-phylogenetic mixture models.

Database	Model	**π**_**p**_	**π**_**q**_	**π**_**r**_
COG	Phylogenetic	0.08	0.87	0.87

COG	Non phylogenetic	0.02	0.16	0.69

OFAM	Non phylogenetic	1.19 × 10^-9^	4.67 × 10^-3^	0.21

TRIBES	Non phylogenetic	0.019	0.074	0.58

π_p _(probability that a non-essential gene is present in a parasitic genome), π_q _(probability that a non-essential gene is present in a non-parasitic genome). π_r _is the probability of presence of essential gene families in both parasitic and non-parasitic genomes.

#### Comparison of 16S rRNA, conditioned logdet and SHOT trees

Table 3 shows the Robinson-Foulds (RF) distance between the trees estimated for each dataset using conditioned logdet distances, either alone or with the mixture models, and the SHOT and 16S rRNA trees. Using the non-phylogenetic mixture model resulted in conditioned logdet trees that were closer to the SHOT tree than when the data were not partitioned. Using the phylogenetic mixture model gave a conditioned logdet tree that was more different from the 16S rRNA tree and the SHOT tree than when the data were not partitioned. The SHOT and 16S rRNA trees are similar.

**Table 3:** Robinson-Foulds distance between pairs of trees estimated using a range of methods

Database	COG	TRIBES	OFAM
CL/SHOT	30	74	89
CL non-phylo/SHOT	22	60	58
CL phylo/SHOT	32	n/a	n/a
**CL/RNA**	**44**	**60**	**63**
**CL non-phylo/RNA**	**46**	**45**	**62**
**CL phylo/RNA**	**46**	n/a	n/a
SHOT/RNA	42	82	74

CL (conditioned logdet); CL non-phylo (conditioned logdet with non-phylogenetic mixture model); CL phylo (conditioned logdet with phylogenetic mixture model); SHOT (SHOT distances and BIONJ); RNA (16S rRNA and PHYML). Distances marked n/a were not calculated because the phylogenetic mixture model was only applied to the COG dataset.

#### Simulation studies

Table 4 shows the results of simulation studies using conditioned logdet, non-phylogenetic and phylogenetic mixture models, with data simulated on a true tree without a parasites clan. The parasites clan is present in all trees estimated using conditioned logdet alone, 78% of the trees estimated from the non-phylogenetic model, and none of the trees estimated from the phylogenetic mixture model.

**Table 4:** Percentage of estimated trees containing parasites clan

Phylogeny estimation method	Trees containing parasites clan (%)
Conditioned logdet	100

Conditioned logdet and non-phylogenetic model	78

Conditioned logdet and phylogenetic model	0

Result of applying the three phylogeny estimation methods to 100 simulated datasets. Shows how many trees contain the parasites clan from conditioned logdet, conditioned logdet and non-phylogenetic mixture model and conditioned logdet and phylogenetic mixture model.

## Conclusions

The parasites clan is unlikely to be an ortholog identification artefact, as it is present in trees estimated from all three databases using conditioned genome reconstruction. The application of the non-phylogenetic mixture model to data from the COG and TRIBES databases generates phylogenetic trees in which the parasites do not form a clan. Thus, it remains plausible that the artefactual parasites clan is caused by heterotachy. However, none of our hybrid methods produced reliable trees.

A potential limitation is that although the 16S tree which we used as a reference tree is a widely used standard tree for bacterial phylogenetics, it may itself have been affected by horizontal gene transfer, and often conflicts with both trees for individual protein coding genes [38], and trees based on the concatenated alignments of many genes [18].

It has been suggested that a lack of models of gene gain and loss is the reason that gene content methods have been unsuccessful [39]. This lack has been partly addressed in several recent studies [23,40,41]. However, these models are not yet likely to give good phylogeny estimates. For example, the phylogenetic mixture model used here gives a higher likelihood to a tree containing a parasites clan than to the 16S rRNA tree [23]. Here, we simulated gene content data under the best available gain-loss model that allows gain and loss rates to change in parasites, on a tree that did not contain a parasites clan. Conditioned logdet alone always estimated an artefactual parasites clan from these data. Using the non-phylogenetic mixture model in conjunction with conditioned logdet resulted in estimating a parasites clan less often. With the phylogenetic mixture model in conjunction with conditioned logdet, the parasites clan was never recovered from the analysis. If it is really the case that the parasites do not form a clan, it therefore seems likely that the gain-loss model used is still an inadequate description of the real process of genome evolution. It may also be the case that small-sample biases in parameter estimation contribute to the problem. In summary, it is possible that more sophisticated models will perform better. Alternatively, there may simply be too little information in gene content data to place the parasites accurately, when so many genes have been lost from them.

The conditioned logdet software and web server we have developed did not deal well with parasites but are statistically sound in the absence of large scale losses. Our method can be expected to give more accurate tree estimates than conditioned logdet with a single conditioning genome. Despite the criticism that conditioned genome reconstruction cannot distinguish between a unique fusion event and several lateral gene transfers [11], conditioned logdet methodology may provide useful information about evolutionary relationships when non-heterotachous, vertical evolutionary models are an adequate approximation for the portion of the tree considered.

## Availability and Requirements

• **Project name**: Conditioned genome reconstruction

• **Project home page**: http://www.liv.ac.uk/~cgrbios/cond_logdet.html

• **Operating system(s)**: Platform independent

• **Programming language**: Perl, CGI and C

• **Other requirements**: None

• **License**: None

• **Any restrictions to use by non-academics**: None

## Authors' contributions

MS implemented the C code. MS, ES and DB developed the methods described in the Supplementary material. AS implemented the web server, analyzed the data, and drafted the manuscript. All authors read and approved the final manuscript.

## Supplementary Material

Additional file 1 **Methods used in conditioned genome reconstruction**. This pdf document contains descriptions of the methods used to calculate conditioned logdet distances and the non-phylogenetic mixture model. Click here for file

Additional file 2 **Further results**. This pdf document contains the results of applying conditioned logdet distances with and without the mixture model to the TRIBES and OFAM databases, and the SHOT and 16S rRNA trees for all three databases. Click here for file
