# Supplementary material for: On the artefactual parasitic eubacteria clan in conditioned logdet phylogenies: heterotachy and ortholog identification artefacts as explanations
Source: BMC Evol Biol. 2010 Nov 9;10:343. doi: 10.1186/1471-2148-10-343 (PMC2992526; doi:10.1186/1471-2148-10-343)
Supplement: Additional file 1 — Methods used in conditioned genome reconstruction. This pdf document contains descriptions of the methods used to calculate conditioned logdet distances and the non-phylogenetic mixture model. [file 1471-2148-10-343-S1.PDF]

Additional file 1 - Methods used in conditioned genome reconstruction.

Ajanthah Sangaralingam, Edward Susko, David Bryant and Matthew Spencer.

August 7, 2010

1 Non-existent distances

Here, we describe the approach we used to deal with the problem of undefined estimated conditioned logdet distances between pairs of genomes, which occurs at a low frequency in real gene content data. We first explain the usual maximum likelihood estimate of a logdet distance, and the circumstances under which this estimate can be undefined. We present evidence suggesting that sampling error is a possible explanation for the problem of undefined distances, although variation in gain and loss rates among gene families can cause similar problems. We then propose a constrained maximum likelihood and pseudocount method which can give us useful distance estimates even when the maximum likelihood estimate is undefined. We have implemented this method in software, along with several less successful alternatives, which we also describe here.

There are possible applications to other logdet distance estimation problems, such as protein logdet, which are currently tackled using pseudocounts. In protein data, the usual cause of undefined distances is states (amino acids) that are completely absent from one of a pair of sequences. This can be addressed using pseudocounts (Thollesson, 2005). For gene content data, the complete absence of a state is not usually the cause of undefined distances, so pseudocounts alone are not sufficient.

We do not pursue the alternative approach of developing a tree estimation method that can handle missing distances, because this would be much more complicated.

Conditioned logdet distances are asymptotically tree-additive and should allow the estimation of a correct phylogeny from data on the presence/absence of gene families. In practice, however, we get a well supported clan of parasites and endosymbionts when this method is applied to bacterial gene content data. One possible explanation for this that there are two categories of genes: a category of genes ('non-essential genes') that have accelerated loss rates in intracellular parasites, endosymbionts, and other organisms that can rely on a host for many metabolic processes; and a category of genes ('essential genes') having the same loss rates in all taxa. This could lead to a kind of heterotachy known to cause problems for many phylogenetic methods (Kolaczkowski and Thornton, 2004). In particular, logdet distances are not necessarily tree-additive when there are categories of genes with different rates of evolution. We discuss the details of a non-phylogenetic

mixture model that we have applied to this method.

1.1 Background

The standard (unconditional) logdet distance between a pair of taxa w and x is

$$d_{wx} = -1/s \log \det([\mathbf{\Pi}^{(w)}]^{-1/2}\mathbf{F}^{(wx)}[\mathbf{\Pi}^{(x)}]^{-1/2}) \tag{1}$$

where s is the number of possible states (e.g. 2 for presence/absence data), $\mathbf{\Pi}^{(i)}$ is a diagonal matrix of state frequencies in taxon i, with diagonal elements $\pi_j^{(i)}$ being the frequency of state j in taxon i, and $\sum_j \pi_j^{(i)} = 1$. $\mathbf{F}^{(wx)}$ is a matrix of pairwise pattern probabilities for taxa w and x, with $\sum_{ij} f_{ij} = 1$.

The conditioned logdet distance between w and x, calculated for genes present in a conditioning genome, is

$$d'_{wx} = -1/2 \log \det([\mathbf{\Pi}'^{(w)}]^{-1/2}\mathbf{F}'^{(wx)}[\mathbf{\Pi}'^{(x)}]^{-1/2}) \tag{2}$$

$\mathbf{F}'^{(wx)}$, $\mathbf{\Pi}'^{(w)}$ and $\mathbf{\Pi}'^{(x)}$ are defined as above, except that they include only those genes present in the conditioning genome. We showed elsewhere (Spencer et al., 2007) that conditioned logdet distances are tree-additive and non-negative, provided that all states have nonzero frequencies and edge lengths are nonzero.

We want to estimate conditioned logdet distances from empirical data. From now on, we will drop the primes because we will always be referring to variables conditional on the presence of gene families in the conditioning genome.

1.2 Maximum likelihood estimates

First, note that the entries in the state probability matrices are determined by the pattern probabilities:

$$\begin{aligned}
\pi_0^{(w)} &= f_{00}^{(wx)} + f_{01}^{(wx)} \\
\pi_1^{(w)} &= f_{10}^{(wx)} + f_{11}^{(wx)} \\
\pi_0^{(x)} &= f_{00}^{(wx)} + f_{10}^{(wx)} \\
\pi_1^{(x)} &= f_{01}^{(wx)} + f_{11}^{(wx)}
\end{aligned} \tag{3}$$

where state 0 represents absence and 1 represents presence. Thus our estimate of the conditioned logdet distance depends only on our estimate of the pattern probability matrix. Furthermore, the determinants of the state probability matrices are always positive if every state has a nonzero probability. Thus, so long as every state has a nonzero probability in both genomes, the logdet distance will be defined provided the pattern probability matrix has a positive determinant. If the determinant of the pattern probability matrix is zero or negative, the estimated distance will be infinite or complex, and thus undefined for practical purposes. We therefore restrict our attention to obtaining an estimated pattern probability matrix with a positive determinant.

The data for any pair of genomes w, x are a matrix of pattern counts:

$$\mathbf{N} = \begin{bmatrix} n_{00} & n_{01} \\ n_{10} & n_{11} \end{bmatrix} \tag{4}$$

where n_{ij} is the number of gene families having presence/absence state i in genome w and j in genome x. If gene families are independent, the log likelihood of these data for a given pattern probability matrix is the multinomial

$$l(\mathbf{F}) = \sum_{ij} n_{ij} \log f_{ij} \tag{5}$$

The maximum likelihood (ML) estimates of the pattern probabilities are then

$$\hat{f}_{ij} = \frac{n_{ij}}{\sum_{ij} n_{ij}} \tag{6}$$

1.3 When ML estimates are unsatisfactory

Occasionally the ML estimate $\hat{\mathbf{F}}$ has a zero or negative determinant. When this happens, the logdet distance is infinite or complex, which makes it difficult to use for phylogeny estimation. Our previous results (Spencer et al., 2007) show that the true pattern probability matrix will have a positive determinant if gene families are independent and identically distributed, and the true unconditioned edge lengths are nonzero and finite. We now consider two possible causes of infinite or complex estimated distances, sampling error and rate variation among gene families. Empirical data show patterns in the frequency of infinite or complex estimated distances that are consistent with sampling error. We show that rate variation among gene families can also result in the true conditioned logdet distances being infinite or complex.

1.4 Sampling error is a possible explanation

Table 1 shows the frequency of infinite or complex conditioned logdet distances in subsets of three ortholog databases, arranged in increasing order of size. Roughly the same taxa are used in each case. The COG subset is the set of all bacterial genomes in COG (Tatusov et al., 2003). In TRIBES (Enright et al., 2003) and OFAM (Goldovsky et al., 2005), we selected genomes with the same species name, but in some cases there were several different strains available, and it was not obvious which one, if any, matched the strain present in COG. In these cases we included all such strains. As the number of gene families in the database increases, the proportion of infinite or complex distances when using the ML estimate of the pattern probability matrix decreases. This is the pattern we would expect to see if the true pattern probability matrices had positive determinants. Thus it seems plausible that sampling error is responsible for the occurrence of infinite or complex distances when using ML estimation. Although these problems occur for only a few pairs of genomes, even one such case prevents us from estimating a tree from a distance matrix using standard methods.

1.5 Will mixture models give zero or negative determinants?

It is likely that different gene families have different gain and loss rates. One way to represent this is as a mixture model in which each gene family belongs to one of several categories, with each category having a different conditional pattern probability matrix. We show here that such a model can result in a marginal pattern probability matrix with a non positive determinant,if the pattern probability matrices for each category are not symmetric.

Table 1: Frequency of problem distances (infinite or complex ML estimates) in three ortholog databases. Databases are arranged in increasing order of size. m and n are the numbers of genomes and gene families in the subsets of each database that we used. $m(m-1)(m-2)/2$ is the number of conditioned logdet distances (m conditioning genomes, $(m-1)(m-2)/2$ distinct pairwise distances among all other genomes for a given conditioning genome), n_p is the number of distances for which the ML conditioned logdet distance was infinite or complex, and p_p is the proportion of distances for which this occurred.

Database	m	n	$m(m-1)(m-2)/2$	n_p	p_p
COG	50	4873	58800	123	2.1×10^{-3}
TRIBES	87	209020	317985	38	1.2×10^{-4}
OFAM	87	308593	317985	20	6.3×10^{-5}

For a 2×2 matrix $\mathbf{A} = \begin{bmatrix} a & b \\ c & d \end{bmatrix}$, the eigenvalues are

$$\begin{aligned} \lambda &= \frac{1}{2}\left[(a+d) \pm \sqrt{(a+d)^2 - 4(ad-bc)}\right] \\ &= \frac{1}{2}\left[(a+d) \pm \sqrt{(a+d)^2 - 4\det\mathbf{A}}\right] \end{aligned} \tag{7}$$

$a, d > 0$,

For pattern probability matrices, we know that $a, b, c, d > 0$. If $\det\mathbf{A} > 0$, then $(a+d) > \sqrt{(a+d)^2 - 4\det\mathbf{A}}$, and both eigenvalues are positive. Thus a 2×2 symmetric matrix with a positive determinant is positive definite. In a mixture model with C categories, the marginal pattern probability matrix is

$$\mathbf{F} = \sum_{i=1}^{C} w_i \mathbf{F}_i \tag{8}$$

where w_i are positive weights that sum to 1, and $\mathbf{F}_i$ is the conditional pattern probability matrix for gene families in the ith class. Under our previous results (Spencer et al., 2007), the true pattern probability matrix for each class is positive definite if it is symmetric. If so, the overall pattern probability matrix is a linear combination of positive definite matrices, and is therefore itself positive definite (Horn and Johnson, 1985, Observation 7.1.3). However, the pattern probability matrices for each category may have positive eigenvalues but not be symmetric (this is likely if different genomes have very different sizes). In such cases, there are sets of pattern probability matrices with positive eigenvalues for which a positive linear combination has a negative determinant. For example,

$$\mathbf{F} = 0.2\begin{bmatrix} 1/3 - \epsilon/3 & \epsilon \\ 1/3 - \epsilon/3 & 1/3 - \epsilon/3 \end{bmatrix} + 0.8\begin{bmatrix} 0.1 - \epsilon/3 & 0.8 - \epsilon/3 \\ \epsilon & 0.1 - \epsilon/3 \end{bmatrix} \tag{9}$$

for all small ϵ, is a positive linear combination of matrices with positive eigenvalues, but has a negative determinant. We do not know how often such cases will occur. The empirical patterns discussed above suggest that they are not common, and there are many mixtures of non-symmetric pattern probability matrices with

positive determinants. For instance, if evolutionary distance is small, it is likely that the expected diagonal entries will be larger than the expected off-diagonal entries, resulting in a positive determinant. Nevertheless, separating gene families into categories when mixtures are suspected may be worthwhile. It may also be possible to prove more about the conditions under which $\mathbf{F}$ will have a positive determinant (see Bryant et al. (2003) for related work on mixtures of pattern probability matrices).

1.6 Constrained maximum likelihood estimates

If sampling error is the cause of undefined distances, then any estimator that gives the ML estimate of the pattern probability matrix when this has a positive determinant will be consistent, because the ML estimate is consistent and will have a positive determinant for infinite sample size. We now consider constrained estimates that have this property.

Suppose we maximize the log likelihood (Equation 5) subject to the constraints $G(\mathbf{F}) = \sum_{ij} f_{ij} - 1 = 0$, $H(\mathbf{F}) = \det \mathbf{F} - d \geq 0$ for some constant d. If the ML solution does not satisfy the determinant constraint, we need to solve

$$\begin{aligned} \frac{\partial l(\mathbf{F})}{\partial f_{ij}} + \lambda_1 \frac{\partial G(\mathbf{F})}{\partial f_{ij}} + \lambda_2 \frac{\partial H(\mathbf{F})}{\partial f_{ij}} &= 0 \\ G(\mathbf{F}) &= 0 \\ H(\mathbf{F}) &= d \end{aligned} \tag{10}$$

with Lagrange multipliers λ_1, λ_2 and

$$\frac{\partial H(\mathbf{F})}{\partial f_{ij}} = \begin{cases} f_{11} & i,j = 0,0 \\ -f_{10} & i,j = 0,1 \\ -f_{01} & i,j = 1,0 \\ f_{00} & i,j = 1,1 \end{cases} \tag{11}$$

For $d = 0$, we can get an analytical solution

$$\begin{aligned} \hat{\pi}_0^{(w)} &= \frac{n_{00} + n_{01}}{\sum_{ij} n_{ij}} \\ \hat{\pi}_1^{(w)} &= \frac{n_{10} + n_{11}}{\sum_{ij} n_{ij}} \\ \hat{\pi}_0^{(x)} &= \frac{n_{00} + n_{10}}{\sum_{ij} n_{ij}} \\ \hat{\pi}_1^{(x)} &= \frac{n_{01} + n_{11}}{\sum_{ij} n_{ij}} \\ \hat{f}_{ij} &= \hat{\pi}_i^w \hat{\pi}_j^x \end{aligned} \tag{12}$$

In other words, the pattern probabilities are the products of the ML estimates of state probabilities in the two taxa. Intuitively, this makes sense because if $\det \mathbf{F} = 0$, the estimated distance is infinite and state probabilities in one taxon are independent of state probabilities in the other.

This does not help us to estimate a finite distance. A variety of ad-hoc methods are possible:

- We could set some small but arbitrary $d > 0$ and attempt to solve Equation 10. We have not been able to do this analytically. If we were able to do this, we could partially justify our choice of d by

selecting the largest d such that a likelihood ratio test does not reject the resulting estimate of $\mathbf{F}$ when compared to the ML estimate, at some specified test size α. We could also use tests based on the estimated conditioned logdet distances (Massingham and Goldman, 2007)

- We could obtain the estimates for $d = 0$, and then use

$$\tilde{\mathbf{F}} = (1-p)\hat{\mathbf{F}} + p\mathbf{A} \tag{13}$$

  for some other matrix $\mathbf{A}$ and some $0 < p < 1$. We pursue this idea because it is simple. It is also consistent provided that $p \to 0$ as $N = \sum_{ij} n_{ij} \to \infty$.

1.7 Constrained ML + pseudocount estimates

Developing the ideas in the last section, we could find the constrained ML estimate $\hat{\mathbf{F}}$ of $\mathbf{F}$ subject to $\det \mathbf{F} \geq 0$. Then if $\det \hat{\mathbf{F}} = 0$, we could add a pseudocount corresponding to one extra observation, divided equally between the 0,0 and 1,1 patterns. This gives the estimate

$$\tilde{\mathbf{F}} = \begin{bmatrix} \hat{f}_{00} & \hat{f}_{01} \\ \hat{f}_{10} & \hat{f}_{11} \end{bmatrix} N/(N+1) + \begin{bmatrix} 1/2 & 0 \\ 0 & 1/2 \end{bmatrix} 1/(N+1) \tag{14}$$

Similar methods are used in protein logdet (Penny et al., 1999). Adding the pseudocount even when $\det \hat{\mathbf{F}} > 0$ is also possible, but makes very little difference in practice. Pseudocounts are often used in Bayesian estimation of multinomial parameters (Agresti, 2002). Pseudocounts are also used in a similar way in non-Bayesian contexts for example, in Wilson's estimate of a binomial proportion (Brown et al., 2002).

1.8 Software

We implemented the methods discussed above in the open-source software `cond_logdet`, which is written in C and is available from `http://www.liv.ac.uk/~matts/genecontent.html`. The distance estimation options are:

- ML logdet distance estimates (`-eM`). This is the default option (although our web server, `http://www.liv.ac.uk/~cgrbios/cond_logdet.html`, uses constrained ML + pseudocount estimates).

- Constrained ML with the constraint $\det \mathbf{F} \geq 0$ (`-eC`). In other words, it returns the ML estimate if $\det \hat{\mathbf{F}} > 0$, and ∞ otherwise. This will only be useful if we have a tree estimation method that can correctly deal with infinite distances. Our current supertree method `BIONJ_COND` (Spencer et al., 2007) is based on BIONJ (Gascuel, 1997). It constructs a tree from a set of m distance matrices, where matrix k is estimated using conditioning genome k and has row and column k missing. We know that we can reconstruct a tree from the set of subtrees each lacking one leaf, but this may not be true if more than one leaf is missing. Unrooted supertree construction is in general more difficult than the special case we have (Steel et al., 2000). We weight distance matrices by the inverse of their variance, assuming that the variance of a pairwise distance is proportional to the distance

- Laplace pseudocount estimates (`-eP`). These do not solve the problem of infinite or complex distances in practice.
- Constrained ML + pseudocount estimates (`-eI`). This is currently the most satisfactory option for real data, because it is simple, consistent and returns a complete distance matrix. This option is used by the web server.
- Return the determinant of the pattern probability matrix rather than the logdet distance (`-eD`). Used for debugging.

The input format is a phylip-style file of gene presence/absence data in each genome. The output is a set of phylip-style distance matrices, one per conditioning genome, concatenated in a single file, and a separate file of conditioning genome sizes. This format can be used directly by `BIONJ_COND`.

2 Non-phylogenetic mixture model

Conditioned logdet distances are asymptotically tree-additive and should allow the estimation of a correct phylogeny from data on the presence/absence of gene families. However, in practice, we get a well supported clan of parasites and endosymbionts when this method is applied to bacterial gene content data. One possible explanation for this that there are two categories of genes: a category of genes ('non-essential genes') that have accelerated loss rates in intracellular parasites, endosymbionts, and other organisms that can rely on a host for many metabolic processes; and a category of genes ('essential genes') having the same loss rates in all taxa. This could lead to a kind of heterotachy known to cause problems for many phylogenetic methods (Kolaczkowski and Thornton, 2004). In particular, logdet distances are not necessarily tree-additive when there are categories of genes with different rates of evolution.

One way to tackle this problem would be to use a mixture model that accounts for the presence of these two classes. If the phylogenetic relationships among genomes are ignored, the gene family presence/absence data can be treated as arising from a binomial mixture model. Although ignoring the dependency among genomes is not strictly correct, it has the advantages of simplicity and not requiring a known tree.

The general form of a mixture model for discrete data with a finite number of categories is

$$P(x; \mathbf{p}, \boldsymbol{\theta}) = \sum_{j=1}^{c} p_j P_j(x; \theta_j) \tag{15}$$

where $P(x; \mathbf{p}, \boldsymbol{\theta})$ is the probability mass function for random variable X, $\mathbf{p} = [p_1, p_2, \ldots p_c]$ is a vector of mixing proportions, $\boldsymbol{\theta}$ is a vector of model parameters, c is the number of categories, $P_j(x;\boldsymbol{\theta}_j)$ is the probability mass function for X conditional on component j with parameters $\boldsymbol{\theta}_j$ (Everitt and Hand, 1981, p. 89).

$X = \{x_{ij}\}$, where

$$x_{ij} = \begin{cases} 0 & \text{gene family } j \text{ absent in genome } i \\ 1 & \text{gene family } j \text{ present in genome } i \end{cases} \tag{16}$$

Treating gene families as independent, the log likelihood for the complete data set is

$$l(X; p, \boldsymbol{\theta}) = \sum_{j=1}^{n} \log[pL_p(x_j; \boldsymbol{\theta}_p) + (1-p)L_r(x_j; \boldsymbol{\theta}_r)] \tag{17}$$

where p is the probability that a gene belongs to the non-essential category, n is the number of gene families, $\boldsymbol{\theta}_p$ is the parameters for the category of genes non-essential in parasites, $\boldsymbol{\theta}_r$ is the parameters for the remaining genes, $L_p(x_j; \boldsymbol{\theta}_p)$ is the likelihood for gene family j conditional on the non-essential category, and $L_r(x_j; \boldsymbol{\theta}_r)$ is the likelihood for gene family j conditional on the essential category. For the essential category, we use a binomial model

$$L_r(x_j; \boldsymbol{\theta}_r) = \pi_r^{n_j}(1-\pi_r)^{m-n_j} \tag{18}$$

where $\pi_r = e^{\beta_0}/(1+e^{\beta_0})$ is the probability that a gene family is present in a genome, conditional on the essential category, parameterized by a logistic model with one parameter β_0 (the log odds of a essential gene being present), m is the number of genomes, and n_j is the number of genomes in which gene family j is present.

For the non-essential category, we add an extra parameter that distinguishes between parasite and non-parasite genomes:

$$L_p(x_j; \boldsymbol{\theta}_p) = \pi_p^{n_{pj}}(1-\pi_p)^{m_p-n_{pj}}\pi_q^{n_{qj}}(1-\pi_q)^{m_q-n_{qj}} \tag{19}$$

where π_p and π_q are the probabilities that a non-essential gene is present in a parasite and a non-parasite genome respectively, m_p and m_q are the numbers of parasite and non-parasite genomes, and n_{pj} and n_{qj} are the numbers of these genomes in which gene family j is present. We model the probabilities of gene presence as

$$\begin{aligned} \pi_p &= \frac{\exp(\beta_q + \beta p)}{1+\exp(\beta_q + \beta p)} \\ \pi_q &= \frac{\exp(\beta_q)}{1+\exp(\beta_q)} \end{aligned} \tag{20}$$

where β_q is the log odds that a non-essential gene is present in a non-parasite genome, and β_q is the difference in log odds of presence between a non-parasite and a parasite genome.

Using Bayes' Theorem (Everitt and Hand, 1981, p.10) and treating p as known, we can obtain an empirical Bayes estimate of the posterior probability that a gene family j is in the non-essential category:

$$P(p|x_j) = \frac{pL_p(x_j; \boldsymbol{\theta}_p)}{pL_p(x_j; \boldsymbol{\theta}_p) + (1-p)L_r(x_j; \boldsymbol{\theta}_r)} \tag{21}$$

3 Merging distance matrices

To combine the distance matrices and conditioned genome sizes from one subset of genes with another we need to merge the distance matrices to give a single distance matrix.

Let $\boldsymbol{\Delta}_{ij}$ be the conditioned logdet distance matrix estimated from data partition $i = \{1, 2\}$ with conditioning genome j. Let n_{ij} be the size of conditioning genome j in partition i. We want to recombine the distance matrices into a single matrix

$$\boldsymbol{\Delta}_j^* = w_{1j}\boldsymbol{\Delta}_{1j} + w_{2j}\boldsymbol{\Delta}_{2j} \tag{22}$$

where w_{ij} is the non-negative weight given to $\mathbf{\Delta}_{ij}$, such that $w_{1j} + w_{2j} = 1$. If $\mathbf{\Delta}_{1j}$ and $\mathbf{\Delta}_{2j}$ are both tree-additive, then any linear combination of them is also tree-additive (Spencer et al., 2007), and will give a consistent method. However, we would like to choose the weights to give a minimum-variance sum and thus improve small-sample performance.

In our modified version (Spencer et al., 2007) of BIONJ (Gascuel, 1997), we assume that $V[\mathbf{\Delta}_j] \propto 1/n_j$ for distances estimated from unpartitioned data with conditioning genome j. We will assume that the constant of proportionality c is the same for both partitions. This may not be true, but simplifies things considerably. We will also assume that the distance matrices $\mathbf{\Delta}_{1j}$ and $\mathbf{\Delta}_{2j}$ are independent (they are estimated from different sets of gene families). Then

$$\begin{aligned} V[\mathbf{\Delta}_j^*] &= V[\mathbf{\Delta}_{1j} + \mathbf{\Delta}_{2j}] \\ &= w_{1j}^2 V[\mathbf{\Delta}_{1j}] + w_{2j}^2 V[\mathbf{\Delta}_{2j}] \\ &= c\left(\frac{w_{1j}^2}{n_{1j}} + \frac{w_{2j}^2}{n_{2j}}\right) \end{aligned} \quad (23)$$

For fixed c, this is minimized at $w_{1j} = n_{1j}/(n_{1j} + n_{2j})$.

We also need to know how to weight combined distance matrices from different choices of conditioning genome j. In the modified version of BIONJ with unpartitioned data, we weighted distance matrices by the sizes n_j of their conditioning genomes (Spencer et al., 2007). With the choice of weights for partitions made above, we have

$$\begin{aligned} V[\mathbf{\Delta}_j] &= \left(\frac{n_{1j}^2}{n_{1j}(n_{1j} + n_{2j})^2} + \frac{n_{2j}^2}{n_{2j}(n_{1j} + n_{2j})^2}\right) c \\ &= \frac{c}{n_{1j} + n_{2j}} \end{aligned} \quad (24)$$

which suggests that $n_{1j} + n_{2j} = n_j$ is the appropriate weight for the combined distance matrix $\mathbf{\Delta}_j^*$.

References

Agresti, A. (2002). *Categorical Data Analysis.* John Wiley and Sons, Hoboken, second edition.

Brown, L. D., Cai, T. T., and DasGupta, A. (2002). Confidence intervals for a binomial proportion and asymptotic expansions. *Annals of Statistics*, 30:160–201.

Bryant, D., Huson, D., Kloepper, T., and Nieselt-Struwe, K. (2003). Distance corrections on recombinant sequences. *Lecture Notes in Computer Science*, 2812:271–286.

Enright, A. J., Kunin, V., and Ouzounis, C. A. (2003). Protein families and TRIBES in genome sequence space. *Nucleic Acids Research*, 31(15):4632–4638.

Everitt, B. and Hand, D. (1981). *Finite Mixture Distributions.* Chapman and Hall, London and New York.

Gascuel, O. (1997). BIONJ: an improved version of the NJ algorithm based on a simple model of sequence data. *Molecular Biology and Evolution*, 14(7):685–695.

Goldovsky, L., Janssen, P., Ahrén, D., Audit, B., Cases, I., Darzentas, N., Enright, A. J., López-Bigas, N., Peregrin-Alvarez, J. M., Smith, M., Tsoka, S., Kunin, V., and Ouzounis, C. A. (2005). Cogent++: an extensive and extensible data environment for computational genomics. *Bioinformatics*, 21(19):3806–3810.

Horn, R. A. and Johnson, C. R. (1985). *Matrix Analysis*. Cambridge University Press, Cambridge.

Kolaczkowski, B. and Thornton, J. (2004). Performance of maximum parsimony and likelihood phylogenetics when evolution is heterogeneous. *Nature*, 431:980–984.

Massingham, T. and Goldman, N. (2007). Statistics of the log-det estimator. *Molecular Biology and Evolution*, 24(10):2277–2285.

Penny, D., Hasegawa, M., Waddell, P. J., and Hendy, M. D. (1999). Mammalian evolution: timing and implications from using the logdeterminant transform for proteins of differing amino acid composition. *Systematic Biology*, 48(1):76–93.

Spencer, M., Bryant, D., and Susko, E. (2007). Conditioned genome reconstruction: how to avoid choosing the conditioning genome. *Systematic Biology*, 56:25–43.

Steel, M., Dress, A. W. M., and Böcker, S. (2000). Simple but fundamental limitations on supertree and consensus tree methods. *Systematic Biology*, 49(2):363–368.

Tatusov, R. L., Fedorova, N. D., Jackson, J. D., Jacobs, A. R., Kiryutin, B., Koonin, E. V., Krylov, D. M., Mazumder, R., Mekhedov, S. L., Sverdlov, A. V., Vasudevan, S., Wolf, Y. I., Yin, J. J., and Natale, D. A. (2003). The COG database: an updated version includes eukaryotes. *BMC Bioinformatics*, 4:41.

Thollesson, M. (2005). LDDist: a perl module for calculating logdet pair-wise distances for protein and nucleotide sequences. *Bioinformatics*, 20(3):416–418.
