# Supplementary material for: On the artefactual parasitic eubacteria clan in conditioned logdet phylogenies: heterotachy and ortholog identification artefacts as explanations
Source: BMC Evol Biol. 2010 Nov 9;10:343. doi: 10.1186/1471-2148-10-343 (PMC2992526; doi:10.1186/1471-2148-10-343)
Supplement: Additional file 2 — Further results. This pdf document contains the results of applying conditioned logdet distances with and without the mixture model to the TRIBES and OFAM databases, and the SHOT and 16S rRNA trees for all three databases. [file 1471-2148-10-343-S2.PDF]

On the artefactual parasitic eubacteria clan in conditioned logdet phylogenies: Additional file 2 — Further results

Ajanthah Sangaralingam, Edward Susko, David Bryant, Matthew Spencer

August 12, 2010

1 COG

Figure S1 shows the 16S tree, and Figure S2 the SHOT tree, for the species in our subset of the COG database.

2 TRIBES

Out of 209020 gene families, we retained only the 16122 that occurred in at least one genome in our dataset. The remainder contain little phylogenetic information, and we were unable to assign gene families to categories if these absent gene families were included. In the phylogenetic tree (Figure S3) estimated from conditioned logdet distances [Spencer et al., 2007], the parasites are found together in a clan with high bootstrap support. Firmicutes have bootstrap support of 89%. The alpha, beta, gamma, and epsilon proteobacteria are close to each other on the tree, and there is 100% bootstrap support for the clan containing alpha, beta, and gamma proteobacteria. The effect of applying the non-phylogenetic mixture model (Figure S4) was that the parasites are no longer found in a clan but are found next to species that are from the same taxonomic group, with high bootstrap support. For example in Figure S4, the firmicutes *Mycoplasma genitalium*, *Mycoplasma pulmonis*, *Mycoplasma pneumonia* and *Ureaplasma urealyticum* are incorrectly placed next to spirochaetes and are not found in the same clan as the other firmicutes. The two *Rickettsia* species are found together next to the non-parasitic alpha proteobacteria. Overall, the tree estimated from the non-phylogenetic mixture model was less well-resolved than the tree estimated from the unpartitioned data. Some groups of bacteria that were found together before the mixture model was applied seem to be partitioned after the model was applied. It should be noted once again as with the COG [Tatusov et al., 1997] dataset the parasites are not found in a clan together, but are found together with other parasites that belong to their correct taxonomic subgroups, but not with the non-parasitic members of their taxonomic groups. Most taxa were correctly placed in the tree estimated using SHOT [Korbel et al., 2002] distances (Figure S5).

3 OFAM

Overall, the tree calculated using conditioned logdet distances (Figure S6) does show the major taxa correctly grouped together, mostly with relatively high bootstrap support. An exception to this is the clan containing the firmicutes which has bootstrap support of 53%. The tree estimated from the non-phylogenetic mixture model contained a parasites clan (Figure S7) with a bootstrap support of 89%, compared to bootstrap support of 95% from the unpartitioned data (Figure S6). For this dataset, the non-phylogenetic mixture model did not result in major changes in tree topology. Most species were correctly placed in the tree estimated from SHOT distances (Figure S8), and in the 16S tree for the strains used in both the TRIBES and OFAM analyses (Figure S9).

References

D. H. Huson, D. C. Richter, C. Rausch, T. Dezulian, M. Franz, and R. Rupp. Dendroscope: an interactive viewer for large phylogenetic trees. *BMC Bioinformatics*, 8:460, 2007.

J. O. Korbel, B. Snel, M. A. Huynen, and P. Bork. SHOT: a web server for the construction of genome phylogenies. 18(3):158–162, 2002.

M. Spencer, D. Bryant, and E. Susko. Conditioned genome reconstruction: how to avoid choosing the conditioning genome. *Systematic Biology*, 56:25–43, 2007.

R. L. Tatusov, E. V. Koonin, and D. J. Lipman. A genomic perspective on protein families. *Science*, 278:631–637, 1997.

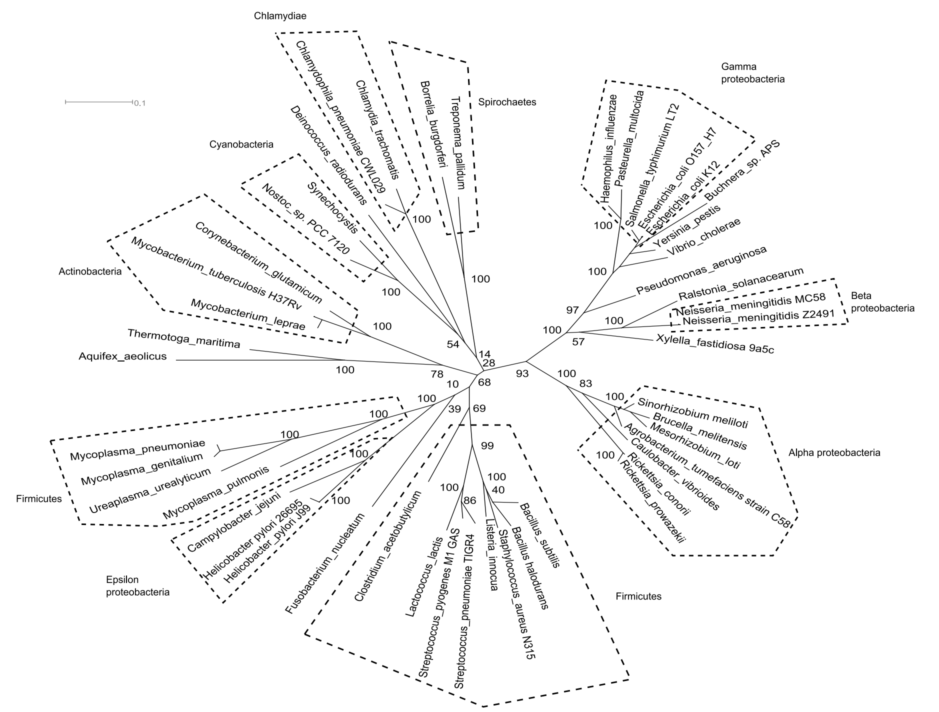

Figure S1: Unrooted radial phylogram for bacterial species from the COG database estimated from 16S rRNA. 1000 bootstrap replicates using PHYML. Tree drawn using Dendroscope [Huson et al., 2007]. Scale bar: 0.1 expected substitutions per site.

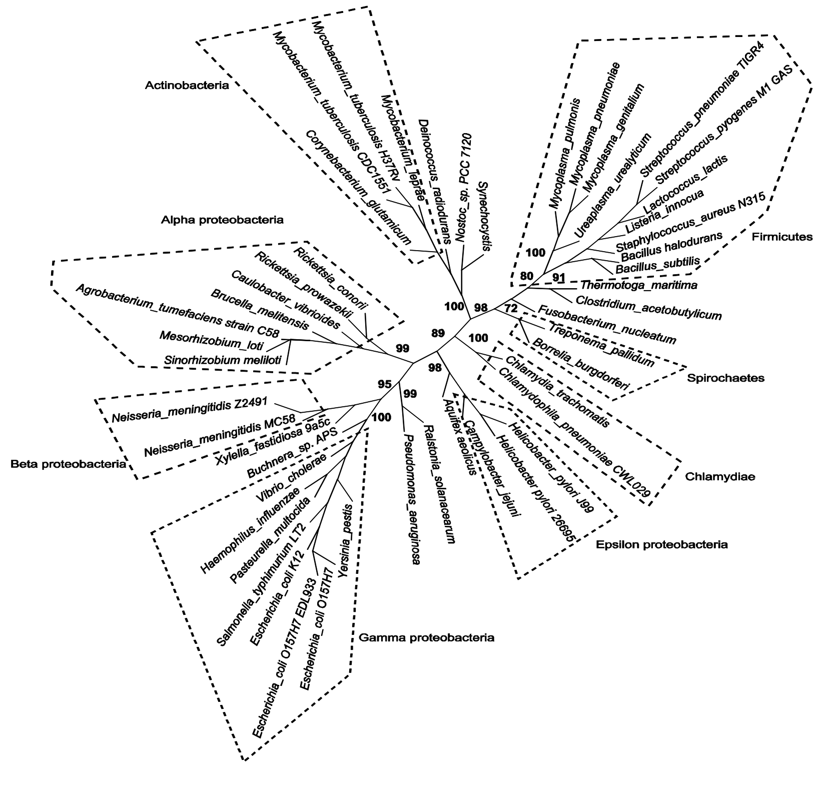

Figure S2: Unrooted radial cladogram for bacterial species from the COG database estimated using the SHOT algorithm and BIONJ. Majority rule, 200 bootstrap replicates using PHYLIP CONSENSE. Tree drawn using Dendroscope [Huson et al., 2007]. Edge lengths not to scale.

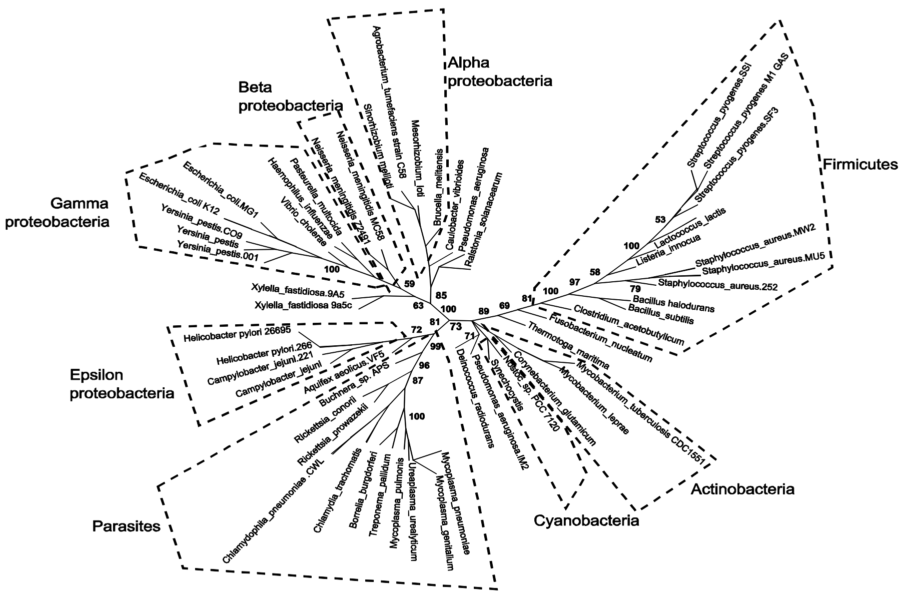

Figure S3: Unrooted radial cladogram for bacterial species from the TRIBES database estimated using conditioned logdet and modified BIONJ. Majority rule consensus, 200 bootstrap replicates using PHYLIP CONSENSE. Tree drawn using Dendroscope [Huson et al., 2007]. Edge lengths not to scale.

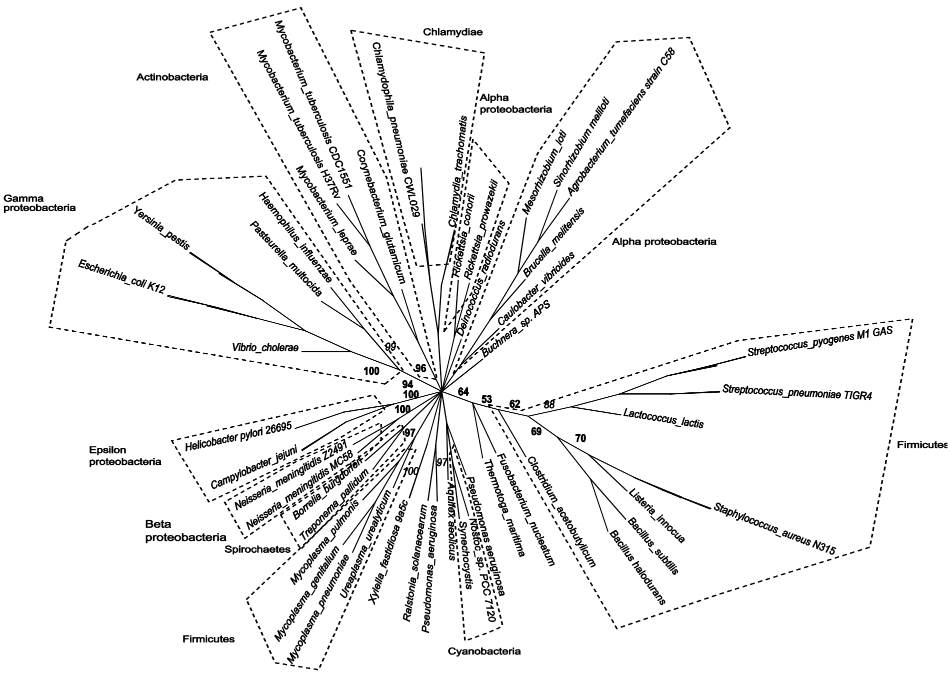

Figure S4: Unrooted radial cladogram for bacterial species from the TRIBES database estimated using a non-phylogenetic mixture model with conditioned logdet and modified BIONJ. Majority rule consensus, 200 bootstrap replicates using PHYLIP CONSENSE. Tree drawn using Dendroscope [Huson et al., 2007]. Edge lengths not to scale.

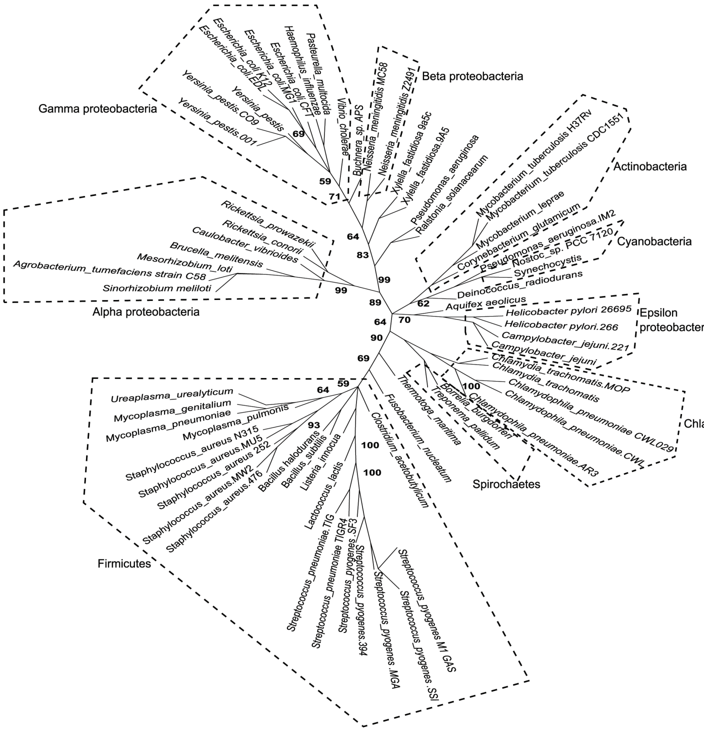

Figure S5: Unrooted radial cladogram for bacterial species from the TRIBES database estimated using the SHOT algorithm and BIONJ. Majority rule consensus, 200 bootstrap replicates using PHYLIP CONSENSE. Tree drawn using Dendroscope [Huson et al., 2007]. Edge lengths not to scale.

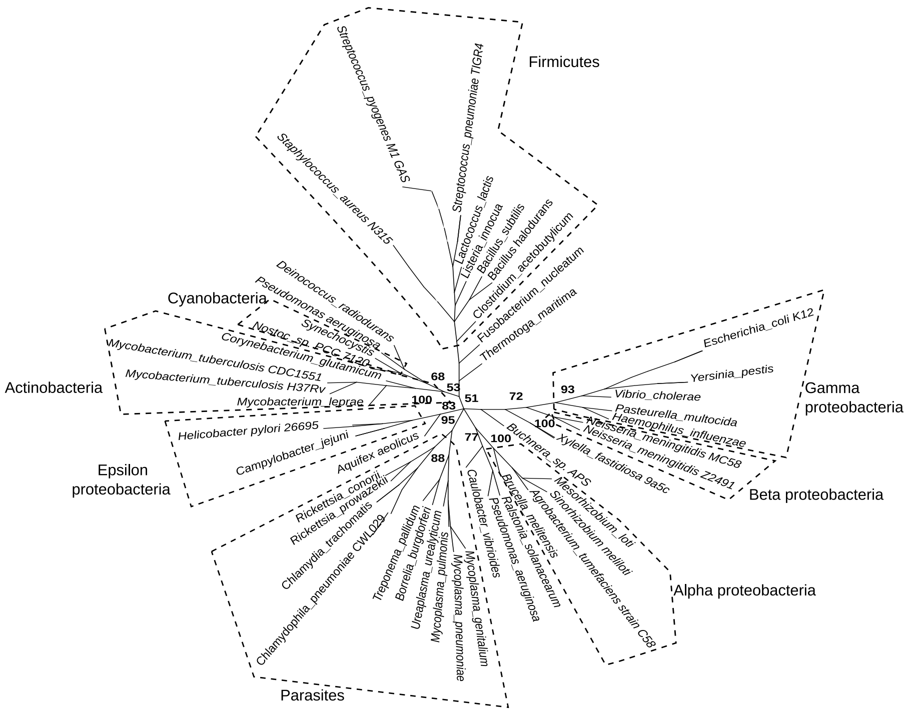

Figure S6: Unrooted radial cladogram for bacterial species from the OFAM database estimated using conditioned logdet and modified BIONJ. Majority rule consensus tree, 200 bootstrap replicates using PHYLIP CONSENSE. Tree drawn using Dendroscope [Huson et al., 2007]. Edge lengths not to scale.

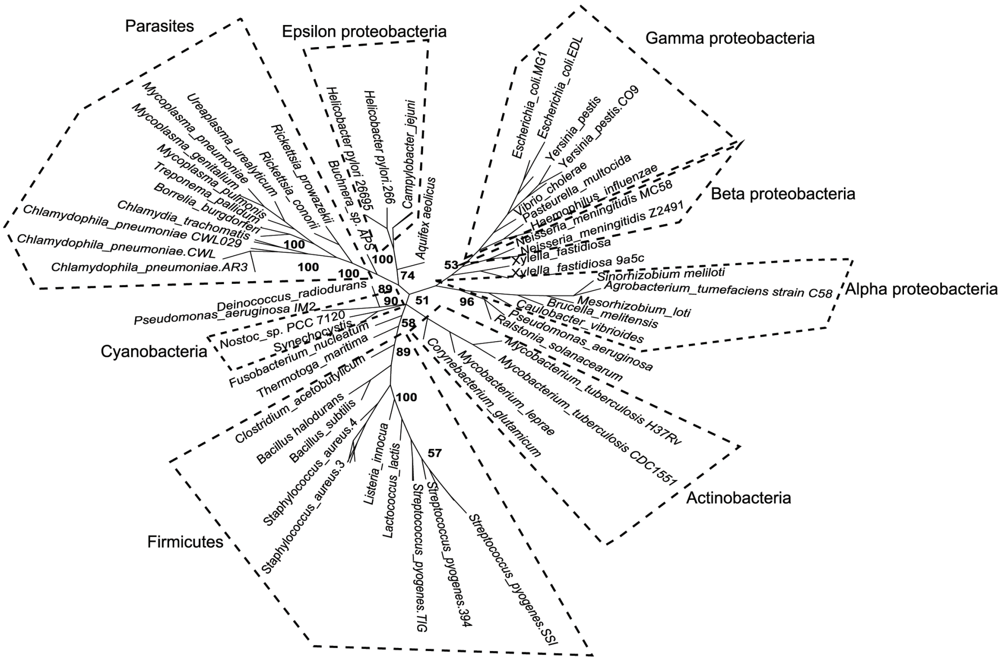

Figure S7: Unrooted radial cladogram for bacterial species from the OFAM database estimated using non-phylogenetic mixture model with conditioned logdet and modified BIONJ. Majority rule consensus tree, 200 bootstrap replicates using PHYLIP CONSENSE. Tree drawn using Dendroscope [Huson et al., 2007]. Edge lengths not to scale.

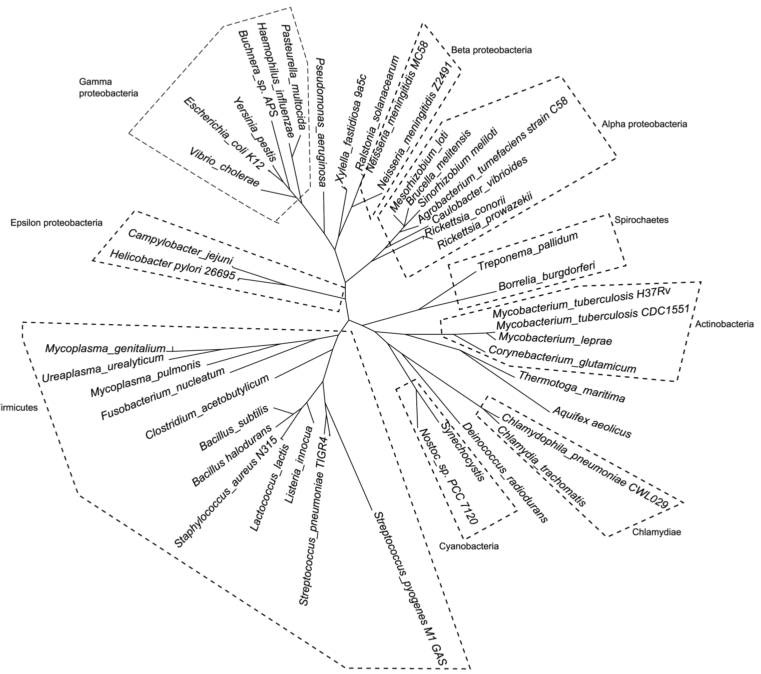

Figure S8: Unrooted radial cladogram for bacterial species from the OFAM database estimated using the SHOT algorithm and BIONJ. Majority rule consensus, 200 bootstrap replicates using PHYLIP CONSENSE. Tree drawn using Dendroscope [Huson et al., 2007]. Edge lengths not to scale.

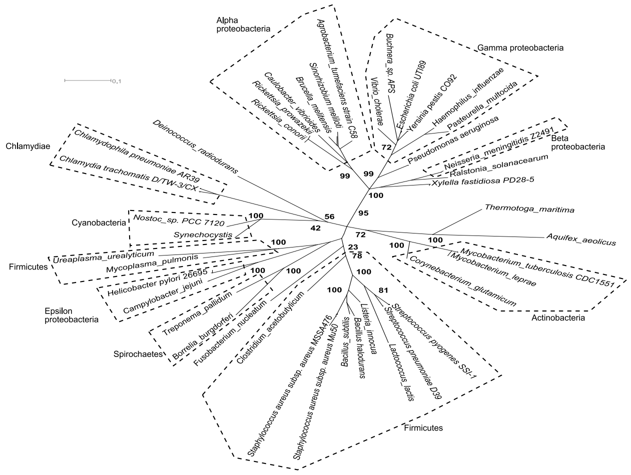

Figure S9: Unrooted radial phylogram for bacterial species from the OFAM and TRIBES databases estimated from the 16S rRNA database. 1000 bootstrap replicates using PHYML. Tree drawn using Dendroscope [Huson et al., 2007]. Scale bar: 0.1 substitutions per site.
